# Interferons in COVID-19: missed opportunities to prove efficacy in clinical phase III trials?

**DOI:** 10.3389/fmed.2023.1198576

**Published:** 2023-05-30

**Authors:** Josef Brzoska, Harald von Eick, Manfred Hündgen

**Affiliations:** Retired, Laupheim, Germany

**Keywords:** COVID-19, coronavirus, clinical trials, interferons—pharmacology, mode of administration

## Abstract

Interferons were repeatedly used in the therapy of COVID-19 due to their antiviral effects. Three recently published randomized controlled clinical phase III trials (WHO SOLIDARITY, ACTT-3, and SPRINTER) missed their primary objectives, i.e., a significant therapeutic effect of interferons was not demonstrated in these studies. In only one randomized controlled phase III trial (TOGETHER), a significant reduction in the hospitalization rate was revealed. Our study analyzes these findings, gives possible explanations for the failure of interferons, provides a proposal on how these agents could be successfully used, and also highlights the limitations of their employment in COVID-19. Interferons are apparently beneficial only if the patients are in the early stage of this disease and when they are usually not hospitalized, i.e., if the patients do not require oxygen support and/or if corticosteroids are not yet indicated. Furthermore, a higher dosage than the one used in the long-term treatment of multiple sclerosis with interferon beta or of chronic viral hepatitis with interferon alpha or lambda should be employed to achieve a better therapeutic effect in COVID-19.

## Introduction

Interferons are naturally produced by virus-infected cells warning other cells against such an attack and inducing an antiviral state in non-infected cells. In addition, interferons activate immune cells to overcome a viral infection. Furthermore, patients with a severe course of a coronavirus infection often reveal a deficient or delayed production of interferons (1–6). Therefore, these agents obtained using biotechnological processes were repeatedly used to treat patients infected with the life-threatening coronaviruses SARS-CoV, MERS-CoV, and SARS-CoV-2. Various interferons have been employed: some of the type I interferons (mainly interferon alfa-2a and alfa-2b and interferon beta-1a and beta-1b) and one type III interferon (interferon lambda-1a). The results obtained were often disappointing (5–10). Three randomized controlled clinical phase III studies concerning the treatment of COVID-19, the WHO SOLIDARITY Trial including the DisCoVeRy Trial (NCT04315948) (11, 12), the ACTT-3 Trial (NCT04492475) (13), and the SPRINTER Trial (NCT04732949) (14), missed their primary objectives (reduction of in-hospital mortality or reduction of time to recovery), i.e., a significant therapeutic effect of the interferons used was not demonstrated in these studies, although there were some hints for efficacy in preceding phase II trials (6–10). Only in the randomized controlled phase III TOGETHER Trial (NCT04727424 and NCT04967430) (15), a significant reduction of the hospitalization rate (defined as the primary objective) was revealed. In this study, we attempt to explain the reasons for the failure of interferons in the phase III trials and make a proposal on how these agents could successfully be used in COVID-19.

## Some important aspects to be considered using interferons in COVID-19

Animal experiments have shown that only a prophylactic administration of interferons or an early beginning of treatment is therapeutically effective in infections with SARS-CoV and MERS-CoV. In contrast, a late start is therapeutically ineffective or can even be harmful due to the induction or enhancement of inflammatory processes by interferon (2, 4, 5). These results have recently been confirmed in further animal models including those with respect to COVID-19 (16–19). The importance of early initiation of interferon treatment could also be demonstrated in clinical phase II trials published in 2020. In a study concerning the treatment of 95 patients suffering from MERS, Arabi et al. (20) found that interferon beta-1b, given subcutaneously in combination with lopinavir/ritonavir, was therapeutically effective only if administered within the first 7 days after the onset of symptoms. When this combination therapy started later, no effect on survival was observed in comparison to a combination of corresponding placebos. Wang et al. (21) examined the association between the use and timing of interferon alfa-2b and clinical outcomes in a retrospective multicenter cohort study of 446 COVID-19 patients. In comparison to the patients not administered interferon, those receiving interferon early (median 2 days after hospital admission) showed reduced in-hospital mortality, whereas late interferon administration (median 8.5 days after hospital admission) was associated with increased mortality [control group: 10/204 (4.9%), early interferon group: 2/216 (0.9%), and late interferon group: 4/26 (15.4%)]. In a randomized study, Davoudi-Monfared et al. (22) treated 92 hospitalized COVID-19 patients with subcutaneously administered interferon beta-1a in combination with standard therapy containing other antivirals or with standard therapy alone. The 46 patients of the interferon arm had symptoms at a median of 10 days (IQR, 8–13) before the start of therapy. In a subgroup analysis, early initiation of interferon treatment resulted in reduced mortality while late start had no significant effects in comparison to standard therapy alone. The concomitant administration of glucocorticoids decreased the positive impact of interferon on mortality. The inhibitory effect of glucocorticoids on the action of interferons had also been observed in other viral infections and in cell culture experiments (3, 23). Concerning a therapeutic effective dosage in acute systemic viral diseases, dose-finding studies had previously been performed in patients suffering from herpes zoster treated with interferon alpha or beta. Only a high-dose therapy with a daily systemic administration of ≥25 MIU interferon given for ca. 5 consecutive days was successful (24).

Accordingly, as already outlined in our preceding studies (5, 24), the successful use of interferons in COVID-19 patients probably requires a consideration of the following three important aspects: (i) an early start of therapy (because interferons do not attack the viruses directly but act primarily by inducing an antiviral state in non-infected cells after binding to specific cell receptors), (ii) no concomitant use of glucocorticoids (as these drugs can block interferon signaling pathways), and (iii) an appropriate dosage and administration route (so that a sufficiently high and persistent interferon level is reached in the infected organs).

## Possible reasons for the failure of interferons in phase III clinical trials

All three aspects mentioned above were neglected in the phase III trials SOLIDARITY (11), ACTT-3 (13), and SPRINTER (14). Treatment was not initiated before the patients were hospitalized and at a mean of 8.6 days (ACTT-3) or 9.5 days (SPRINTER) after the onset of disease symptoms. Respiratory support was required in 76, 84, or 100% of the patients recruited in the SOLIDARITY, ACTT-3, or SPRINTER trials, respectively. Furthermore, a high percentage of patients received systemic corticosteroids at baseline (SOLIDARITY: 59%; ACTT-3: percentage was not stated but corticosteroids were not generally prohibited; SPRINTER: 87%). Interferon beta-1a was administered subcutaneously at a dose of 44 mcg (12 MIU) given on days 1, 3, and 6 (SOLIDARITY) or on days 1, 3, 5, and 7 (ACTT-3), respectively. This therapeutic regimen approximately corresponds to that used per week for long-term treatment of multiple sclerosis with interferon beta-1a. In the SPRINTER trial, the patients received 15.6 MIU interferon beta-1a or placebo by daily inhalation for up to 14 days (Table 1). The dosages used in these studies or local treatment, respectively, are probably not adequate in patients with an advanced stage of COVID-19. Moreover, with non-PEGylated formulations, a daily administration is necessary to achieve a persistent interferon serum level, which is probably important for a therapeutic effect in acute systemic viral diseases (5, 24).

**Table 1:** Interventions and study population in the clinical phase III trials with interferons in COVID-19.

**Clinical phase III trial**	**WHO SOLIDARITY**		**ACTT-3**		**SPRINTER**		**TOGETHER**	
	**Interferon arm**	**Control arm**	**Interferon arm**	**Control arm**	**Interferon arm**	**Control arm**	**Interferon arm**	**Control arm**
**Interventions**								
Treatment (IFN: interferon; SOC: standard of care)	IFN-beta-1a (+ Lopinavir)	SOC (+ Lopinavir)	IFN-beta-1a + Remdesivir	Placebo + Remdesivir	IFN-beta-1a + SOC	Placebo + SOC	IFN-lambda-1a (PEGylated)	Placebo
Interferon dosage	44 μg (12 MIU)	–	44 μg (12 MIU)	–	2 × 7.8 MIU/d	–	180 μg	–
Interferon or placebo administration	Day 1, 3, 6	–	Day 1, 3, 5, 7	Day 1, 3, 5, 7	Day 1–14	Day 1–14	Day 1	Day 1
**Study population**								
Number of patients evaluated	2,144	2,147	487	482	309	314	931	1,018
Mean or median age (years)	50–69	50–69	58.3	59.1	52.0	53.7	43	43
Number of males, females	1,342, 802 (62.6%, 37.4%)	1,331, 816 (62.0%, 38.0%)	297, 190 (61.0%, 39.0%)	266, 216 (55.2%, 44.8%)	203, 106 (65.7%, 34.3%)	208, 106 (66.2%, 33.8%)	400, 531 (43.0%, 57.0%)	436, 582 (42.8%, 57.2%)
Mean duration (days) of symptoms before study entry	(not stated)	(not stated)	8.7	8.5	9.6	9.5	3.3	3.3
Hospitalization at study entry	Yes	Yes	Yes	Yes	Yes	Yes	No	No
Number of patients requiring respiratory support at study entry	1,641 (76.5%)	1,639 (76.3%)	403 (82.8%)	414 (85.9%)	309 (100%)	314 (100%)	0 (0%)	0 (0%)
Number of patients receiving corticosteroids at study entry (or during study)	1,232 (57.5%)	1,286 (59.9%)	(not stated) (corticosteroids not excluded)	(not stated) (corticosteroids not excluded)	267 (86.4%)	275 (87.6%)	0 (>10 mg prednisone)	0 (>10 mg prednisone)

A confirmation of the importance of the aforementioned three aspects is provided by a recently published clinical trial conducted in Hong Kong with 212 patients at high risk of clinical deterioration (25). Similar to the ACTT-3 trial, the patients of the “Hong Kong trial” received a combination of remdesivir plus interferon beta or remdesivir alone (control). However, treatment already started at a median of 3 days (IQR, 2–4) after the onset of clinical symptoms. Only one-third of the patients required oxygen therapy. Corticosteroids (in a “stress dose”) were given to only 22.2% of the interferon and to 36.5% of the control patients. Furthermore, in the Hong Kong trial, interferon beta-1b was administered subcutaneously at a dose of 16 MIU daily for 5 days (for the long-term treatment of multiple sclerosis, interferon beta-1b is used at a lower dosage of 8 MIU given every other day only). The primary endpoint, i.e., the time to complete alleviation of COVID-19 symptoms, was significantly shorter in the combination than in the control group (4 vs. 6.5 days). Moreover, several secondary endpoints of the Hong Kong trial showed distinct differences.

In the SPRINTER trial (14), all 623 patients received oxygen therapy but merely via nasal prongs or masks (the WHO ordinal scale 4). Subjects suffering from a more advanced stage of COVID-19 were not included. Although there was some reduction in the relative risk of progression to severe disease or death in the patients treated with interferon, there was no difference between patients under interferon and those under placebo regarding the primary endpoints (time to hospital discharge and time to recovery) (Table 2). In contrast, the corresponding phase II trial with 98 patients had revealed significant differences between the interferon and the placebo group regarding the improvement of COVID-19 (26). In addition to statistical chance, other reasons might be responsible for this discrepancy. In the phase II trial, approximately one-third of patients had not required oxygen therapy at the baseline (the WHO ordinal scale 3 or less). Furthermore, an improved standard of care (SOC) regarding COVID-19 patients could have diminished the differences between interferon and placebo treatment in the phase III trial. It will be interesting to learn the results of a clinical trial (COVERAGE France) using a nebulized interferon beta-1b in one of the intervention arms (NCT04356495). Here non-hospitalized patients were treated, and the daily application of 9.6 MIU was already started within 7 days after the onset of clinical symptoms.

**Table 2:** Primary (P) and some secondary (S) efficacy endpoints in the clinical phase III trials with interferons in COVID-19.

**Clinical phase III trial**	**Efficacy endpoint**	**Result**	
		**Interferon arm**	**Control arm**
WHO SOLIDARITY	Probability of in-hospital death at 28 days (P)	14.7% (*n*_total_ = 2,144)	12.4% (*n*_total_ = 2,147)
	Probability of in-hospital death at 28 days in patients without mechanical ventilation (S)	12.3% (*n*_total_ = 2,000)	10.7% (*n*_total_ = 2,011)
	Probability of in-hospital death at 28 days in patients with mechanical ventilation (S)	47.9% (*n*_total_ = 144)	37.5% (*n*_total_ = 136)
ACTT-3	Median time (days) to recovery to ordinal scale ≤ 3 (P)	5.0	5.0
	Mortality within 28 days in patients not requiring high flow oxygen or non-invasive ventilation (S)	14/452 (3.1%)	12/448 (2.7%)
	Mortality within 28 days in patients requiring high flow oxygen or non-invasive ventilation (S)	7/35 (20.0%)	4/34 (11.8%)
SPRINTER	Median time (days) to hospital discharge (P)	7.0	8.0
	Median time (days) to recovery to no limitation of activity (P)	25.0	25.0
	Number of patients progressing to severe disease or death within 35 days (S)	33/309 (10.7%)	45/314 (14.3%)
	Mortality within 35 days (S)	14/309 (4.5%)	17/314 (5.4%)
TOGETHER	Number of patients requiring hospitalization or >6 h in an emergency room within 28 days (P)	25/931 (2.7%)	57/1,018 (5.6%)
	Mortality due to COVID-19 within 28 days (S)	1/931 (0.1%)	4/1,018 (0.4%)

## Deterioration of COVID-19 by late administration of interferons

In animal models regarding the life-threatening coronavirus diseases, a late administration of interferons has a detrimental effect (see above). Although there are some differences between the animal models and the corresponding human diseases (4, 5), a negative effect of interferon treatment was also observed in the phase III studies if the patients suffered from an advanced stage of COVID-19 at the baseline (Table 2). In the SOLIDARITY trial (11) with four treatment and matching control arms, 4,291 patients were given interferon (plus lopinavir) or treated with lopinavir/SOC alone (control). In the 280 patients who required mechanical ventilation, the probability of in-hospital death at 28 days was higher in the interferon beta-1a group than in the control group (47.9 vs. 37.5%), and in the 4,011 patients without mechanical ventilation, almost no difference as to mortality was determined between these two groups. In the ACTT-3 trial (13), the therapeutic effect of a combination of remdesivir plus interferon beta-1a vs. remdesivir plus placebo was determined in 969 patients. Among the 69 patients who required non-invasive ventilation or high-flow oxygen (ordinal score 6) at the baseline, mortality was 20.0% in the interferon and 11.8% in the placebo group. Fewer patients under interferon than under placebo recovered within 28 days: 16/35 (46%) vs. 27/34 (79%). There was almost no difference between interferon and placebo patients with less severe disease (ordinal score 5 or 4). Accordingly, patients suffering from COVID-19 in a late stage should not be treated with interferons.

## Positive effects in COVID-19 by early administration of interferons

As outlined above, several animal experiments and certain clinical phase II trials have provided some evidence that only an early start of interferon administration after the onset of clinical symptoms can be therapeutically effective in COVID-19. Out of corresponding phase III studies, data are available for the TOGETHER trial (15). In this study with several treatment and matching placebo arms (NCT04727424 and NCT04967430), patients at risk to develop a severe course of COVID-19 (e.g., patients over 50 years, patients with certain diseases, such as diabetes or arterial hypertension) were treated within 7 days after the symptom onset. None of them was hospitalized, had an acute respiratory condition, or received systemic glucocorticoids >10 mg per day equivalent to prednisone. In the arm investigating the therapeutic effect of interferon, the patients were treated with a single subcutaneous injection of PEGylated interferon lambda-1a in a dose of 180 mcg (equivalent to a dose of ca. 3–5 MIU of a non-PEGylated formulation given thrice weekly for 1 week) (Table 1). In the interferon arm, 25 of 931 patients (2.7%) had to be hospitalized or spent more than 6 h in an emergency room compared to 57 of 1,018 patients (5.6%) in the matching placebo arm. Only one patient died in the interferon group compared to four patients in the placebo group (Table 2).

Interestingly, two preceding corresponding phase II trials using the same dosage of interferon lambda-1a or placebo differed in their results (27, 28). In both studies, treatment started within 7 days after the onset of clinical symptoms. However, patients with a good prognosis were included. In one of these two studies with 60 outpatients, a significant acceleration of viral clearance was determined in the interferon arm (27). In the other clinical trial with 120 outpatients, no effect of interferon lambda was found regarding viral shedding and improvement of symptoms (28).

The dosage used in these studies corresponds to that employed for the long-term treatment of chronic viral hepatitis. This dosage is barely adequate for the therapy of an acute systemic viral disease as discussed elsewhere (5, 24). The positive results obtained in the aforementioned Hong Kong trial (25) also suggest that the dosage of PEGylated interferon lambda-1a should be increased to achieve a higher efficacy in COVID-19 although at the cost of more side effects. Moreover, in order to obtain a significant difference between the interferon and control groups, i.e., to see a greater therapeutic effect of interferon, the inclusion criteria in a further clinical study should be more restricted, i.e., only patients at high risk could be enrolled, e.g., age at least 60 rather than 50 years, combined with at least one other risk factor.

## How to use interferons for a successful treatment of COVID-19: a proposal

What kind of approach should be employed using interferons in the treatment of COVID-19? Out of the type I interferons, interferon beta seems to be superior to interferon alpha for the treatment of acute viral infections because *in vitro* interferon beta has a higher antiviral activity (5, 6), and patients who develop a severe form of COVID-19 often have autoantibodies to interferon alpha but rarely to interferon beta (29). However, a limitation of interferon beta is the fact that it has to be administered by the intravenous route if relatively high serum levels are required. This condition must probably be fulfilled to achieve a therapeutic effect in an acute systemic viral infection such as COVID-19 (5, 24).

Naturally produced type III interferons are regarded as a front-line defense at the mucosal barriers without stimulating systemic inflammation, while type I interferons are probably generated by the body if the viruses escape this local control (4, 30). In comparison to type I interferons, type III interferons have rather anti-inflammatory than pro-inflammatory properties, which make them attractive agents for the treatment of COVID-19 (4, 30–32). However, receptors for type I interferons are commonly expressed in virtually all types of nucleated cells, whereas those for type III interferons are only present in epithelial cells and in a few other cell types (4, 30, 31). This restriction could reduce the overall efficacy of interferon lambda in COVID-19 (31), especially in more advanced stages. Taken together, it remains an open question about which interferon is most appropriate for the treatment of COVID-19.

Regarding the time window, dosage, and administration route of interferons in COVID-19, some general conclusions can be drawn from the results obtained in the different clinical studies. A daily local application (e.g., by inhalation or spray) should be initiated within the first 3–5 days after the onset of clinical symptoms when the patients are usually not yet hospitalized. The objective of this kind of treatment would be to prevent moderate/severe pneumonia and/or hospitalization in patients at risk for these events. The advantage of such a local interferon administration is that relatively low doses can be used to achieve a sufficient interferon level in the (upper) respiratory tract and (systemic) side effects can generally be avoided (5).

When an interferon treatment starts later than symptom day 5 or if no improvement of the disease is observed at that time, a daily systemic administration of interferon is probably necessary for a successful outcome. In high-risk patients, such a therapy may be started right at the onset of disease symptoms. Using a PEGylated formulation, a single interferon dose seems to be sufficient. The dosage should be near the maximum tolerated dose (MTD), i.e., higher than the dosage used in the long-term treatment of multiple sclerosis with interferon beta or that of chronic viral hepatitis with interferon alpha or lambda, respectively. Otherwise, a combination of interferon with other antivirals is probably required for the successful treatment of COVID-19 (24). Starting this more intensive therapy should not be later than 6 to 8 days after the onset of symptoms when glucocorticoids are not yet indicated, in any case before (high flow) oxygen therapy is needed. Moreover, the interferons should not be administered beyond symptom days 10–12 (PEGylated formulations beyond symptom days 6–8) in order to minimize the risk of a possible deterioration of COVID-19 since especially type I interferons can induce or reinforce inflammatory processes which are characteristic for the severe stage of this disease (1–5).

## Discussion

Our analysis reveals that important aspects were not considered in the disappointing clinical phase III trials using interferons in COVID-19: (i) an early start of therapy, (ii) no concomitant use of glucocorticoids, and (iii) an appropriate dosage and administration route. The observance of these aspects appears to be essential for the successful therapeutic employment of these agents in this disease. Due to their antiviral actions, interferons will possibly continue to play a role in the therapy of COVID-19. The availability of vaccines does not eliminate the demand for an effective therapy because even vaccinated people can get infected with SARS-CoV-2, and some of them, especially immunocompromised individuals, show a severe course of COVID-19. Furthermore, several people are unable or unwilling to be vaccinated (33–35). The use of interferons may be an option to prevent a moderate or severe course of COVID-19 in patients at risk. With our proposal (see above), we make some distinct suggestions on how these agents could/should be administered in this disease to achieve a successful clinical outcome. However, before choosing interferons as agents for the treatment of COVID-19, further clinical studies have to be performed to prove whether our proposed approach is suitable and practical. These trials, especially those in high-risk patients, have to be currently performed with vaccinated subjects and/or with a control group receiving one of the drugs presently available and approved for treating COVID-19 (33–35). Such trials would also reveal how appropriate and competitive interferons are in comparison to other agents in this disease. The advantage of interferons is the fact that their antiviral effect is not virus-specific. However, patients in an advanced stage of COVID-19, i.e., those requiring oxygen therapy, are not suitable for interferon treatment.

In some studies, cytokine profiles of COVID-19 patients have been determined as potential prognostic and therapeutic markers (36, 37). Excessive production of inflammatory cytokines, such as IL-6, has been found in severe cases of this disease (3, 38, 39). The levels of these cytokines might be used to modify our general recommendations regarding the time point for the cessation of interferon treatment and for the start, dosage, and duration of corticosteroid administration or anti-cytokine therapy, respectively (3, 36–38). Furthermore, also other laboratory parameters, immuno-histochemical markers, chest computed tomography imaging patterns, or further characteristics predictive for a severe course of COVID-19 (39, 40) might be helpful to decide whether an interferon treatment can be initiated or shall be discontinued, respectively.

According to their mechanism of action (see above), interferons were also used rather prophylactically than therapeutically in viral infections including COVID-19. Some of these clinical studies showed promising but eventually not convincing results (41, 42). Various trials (e.g., NCT04534725, NCT04552379, and NCT05485584) regarding the prevention of a SARS-CoV-2 transmission by interferon administration to persons with contact to COVID-19 patients are still in progress or have recently been completed. It remains to be seen whether the dosages used in these trials are appropriate and effective and whether the benefits outbalance the discomfort and risks of such prophylaxis. In any case, it is clear that vaccination and general hygienic measures will still remain the major means to reduce the number of SARS-CoV-2 transmissions and that of severe courses of this viral infection (33).

## Data availability statement

The original contributions presented in the study are included in the article/supplementary material, further inquiries can be directed to the corresponding author.

## Author contributions

JB made the literature search and wrote the first draft of the manuscript. This draft was intensively discussed with HE and MH and changed according to their comments. All authors contributed to the article and approved the submitted version.
